# DownScaleBench for developing and applying a deep learning based urban climate downscaling- first results for high-resolution urban precipitation climatology over Austin, Texas

**DOI:** 10.1007/s43762-023-00096-9

**Published:** 2023-05-31

**Authors:** Manmeet Singh, Nachiketa Acharya, Sajad Jamshidi, Junfeng Jiao, Zong-Liang Yang, Marc Coudert, Zach Baumer, Dev Niyogi

**Affiliations:** 1 Jackson School of Geosciences, The University of Texas at Austin, Austin, 78712 TX USA; 2 Indian Institute of Tropical Meteorology, Ministry of Earth Sciences, Pune, 411008 MH India; 3 IDP in Climate Studies, Indian Institute of Technology Bombay, Mumbai, 400076 MH India; 4 CIRES, University of Colorado Boulder, NOAA/Physical Sciences Laboratory, Boulder, 80309 CO USA; 5 Department of Agronomy, Purdue University, West Lafayette, 47906 IN USA; 6 Department of Civil, Architectural, and Environmental Engineering, Cockrell School of Engineering, The University of Texas at Austin, Austin, 78712 TX USA; 7 Office of Sustainability, City of Austin, Austin, 78712 TX USA; 8 Oden Institute for Computational Engineering and Sciences, The University of Texas at Austin, Austin, 78712 TX USA

**Keywords:** Urban downscaling, Deep learning, Smart city, Austin, DownScaleBench, Urban meteorology

## Abstract

Cities need climate information to develop resilient infrastructure and for adaptation decisions. The information desired is at the order of magnitudes finer scales relative to what is typically available from climate analysis and future projections. Urban downscaling refers to developing such climate information at the city (order of 1 – 10 km) and neighborhood (order of 0.1 – 1 km) resolutions from coarser climate products. Developing these higher resolution (finer grid spacing) data needed for assessments typically covering multiyear climatology of past data and future projections is complex and computationally expensive for traditional physics-based dynamical models. In this study, we develop and adopt a novel approach for urban downscaling by generating a general-purpose operator using deep learning. This ‘DownScaleBench’ tool can aid the process of downscaling to any location. The DownScaleBench has been generalized for both in situ (ground- based) and satellite or reanalysis gridded data. The algorithm employs an iterative super-resolution convolutional neural network (Iterative SRCNN) over the city. We apply this for the development of a high-resolution gridded precipitation product (300 m) from a relatively coarse (10 km) satellite-based product (JAXA GsMAP). The high-resolution gridded precipitation datasets is compared against insitu observations for past heavy rain events over Austin, Texas, and shows marked improvement relative to the coarser datasets relative to cubic interpolation as a baseline. The creation of this Downscaling Bench has implications for generating high-resolution gridded urban meteorological datasets and aiding the planning process for climate-ready cities.

## Introduction

High-resolution datasets at the neighborhood or sub-km spatial scales are desired for understanding urban climate and developing climate service applications. Spatial resolution indicates how detailed and representative a map or image is. Coarse spatial resolution in an urban context would be data or map with a limited level of detail (typically of the orders of 10s km); the high-resolution data variables have elements typically needed for neighborhood scale analysis. In the context of city planning, high-resolution data can be helpful for mean climate attributes, as it can help city planners make more informed decisions about adaptation strategies (Bixler et al., 2022). For example, climate data with a high level of detail can help city staff identify areas that are most likely to flood or have exceptional heat. It can also help neighborhoods develop strategies that can help them make more equitable choices about future climate patterns. High-resolution data, in general, can be more useful in urban decision-making, analysis for energy use and a host of essential activities than coarse spatial resolution. Note that for most climatological, long-term analyses, airport observations are taken as indicative of the city. Additionally, reanalysis products are 30-100 km and suitable for capturing large-scale dynamics but not for local scale decision-making or important assessments (Tewari et al., 2023). Additionally, studies such as Berne et al. (2004); Ward et al. (2018) discuss the relative importance of spatial and temporal scales for urban hydroclimatology. The high-resolution datasets at the street scale of less than 500 m are desired important for community education, insurance claims, urban ecology and air pollution health studies.

Downscaling is necessary because global climate models, which are used to simulate and predict future climate, have a low spatial resolution. Different downscaling approaches are used in urban studies (Smid & Costa, 2018). Downscaling approaches are postprocessing techniques that can be categorized under two overarching themes statistical and dynamical approaches. Dynamical downscaling uses high-resolution regional climate or numerical weather prediction models to simulate the weather over a smaller domain at a fine spacing/scale. This approach can provide local dynamical feedbacks about local-scale climate conditions. However, dynamical downscaling is computationally intensive and requires a high level of expertise to set up and run the models with appropriate domain size and boundary conditions. Statistical downscaling is often therefore used to improve spatial resolution when a reference baseline dataset is available. This method can be good at capturing the local climate conditions important for city planning. Still, it depends on the availability of high-quality historical climate data and may not be able to accurately capture the effects of climate change. An example of a statistical technique would be developing a relationship using a large-scale climate product and then assuming that relation holds at a local scale and generating high-resolution fields.

Recently more sophisticated statistical approaches are available: for example, change detection method (Hu et al., 2019), Support Vector Machine-Probabilistic Global Search (Njoku et al., 2002), and artificial intelligence (AI). These statistical approaches have gained popularity in recent years due to their ability to upscale and downscale meteorological parameters (e.g., K-Means, Neural network) and due to the relatively quick execution and computational needs compared to the dynamical downscaling methodology. One such statistical techniques involves the convolutional neural networks (CNNs), which is a deep learning framework that consist of a series of convolutional layers that: (i) slide along inputs (as multidimensional arrays), (ii) assign learnable weights, and biases to each neuron, and (iii) generates the featured output map (Ghosh, 2010; Aloysius & Geetha, 2017). Given CNNs ability to learn the patterns from gridded datasets, they have been used in several downscaling approaches (e.g., Gu et al. (2015); Xu et al. (2020)).

### Study objective

**Fig. 1:**
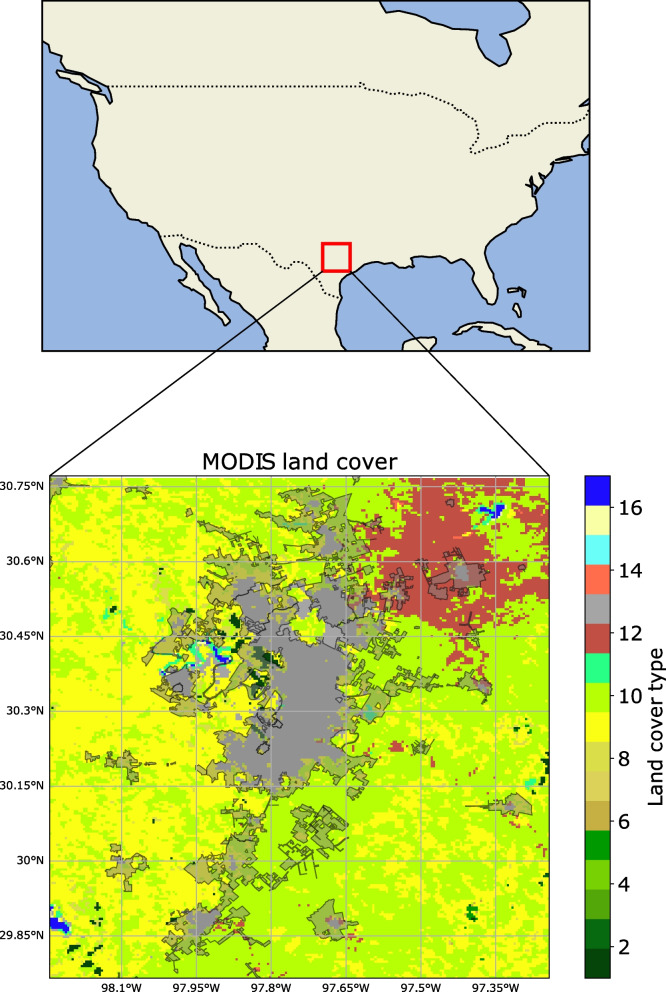
Location of Austin, Texas in the USA. Urban downscaling is performed over a 3 $^{\circ }$ X 3 $^{\circ }$ box (29-32N,96-99W) centered over Austin. The 3 $^{\circ }$ X 3 $^{\circ }$ box is shown as land-use land-cover map over Austin from the MCD12Q1.061 MODIS Land Cover Type Yearly Global 500m using Annual International Geosphere-Biosphere Programme (IGBP) classification. The numbers correspond to the following classes: 1 - Evergreen Needleleaf Forests, 2 - Evergreen Broadleaf Forests, 3 - Deciduous Needleleaf Forests, 4 - Deciduous Broadleaf Forests, 5 - Mixed Forests, 6 - Closed Shrublands, 7 - Open Shrublands, 8 - Woody Savannas, 9 - Savannas, 10 - Grasslands, 11 - Permanent Wetlands, 12 - Croplands, 13 - Urban and Built-up Lands, 14 - Cropland/Natural Vegetation Mosaics, 15 - Permanent Snow and Ice, 16 - Barren, 17 - Water Bodies

The motivation for this work stems from discussions with researchers and city staff working on climate projects in Austin, Texas. Several city-based operations need high-resolution climate information. Currently, the city of Austin is developing a climate projection that can be used for different sustainability operations. For this purpose, the available data is typically from reanalysis or satellite gridded fields and needs to be downscaled. The location of Austin is shown in Fig. 1. As the capital of Texas, United States and the largest city of Travis County in terms of area and population. Since 2010, it has been one of the fastest-growing major American cities. Austin’s population was estimated at 961,855 in the most recent census in 2020.

Development of the urban precipitation high-resolution data/ climatology is important for a variety of planning as well as water resources and disaster response activities. In addition, such information is essential for developing infrastructure as part of the smart city framework (Anthopoulos, 2017). Cities seek to develop climate resiliency and sustainability strategies for which urban scale (i.e., spatial scale order of 1km grid spacing) and climatic datasets are needed (González et al., 2021). There are popular climate data available from international and coordinated assessments that have resulted in the Reanalysis and climate model outputs (e.g., IPCC/CMIP6 (Meehl et al., 2000), or ERA5 (Hersbach et al., 2020)); however, their grid spacing is relatively coarse (order of 10s to 100s of km grid) (Hersbach et al., 2020). Projecting the future state of the atmosphere has been made possible using numerical models (Yang et al., 2016). Despite significant improvements to the numerical models in the last decade, the limitation in the computational power and numerical stability (, 2009) mean the global climate model and reanalysis outputs are generated at coarse spatial resolution. While adequate for modeling mesoscale processes and weather forecasting (Sha et al., 2020), this resolution, as discussed, is not optimal for capturing spatial variability of environmental and climate variables in a heterogeneous environment and complex terrains (Schumacher & Rasmussen, 2020). As a result, cities such as Austin, are generally represented by a single or similar small number of grids from the climate reanalysis fields. The climatology that emerges from such large-scale fields is of limited use for city-scale operations requiring information at a much higher spatio-temporal resolution. City departments need such information to understand local vulnerabilities, assess infrastructure planning needs, and develop resiliency plans considering equity and adaptive options available. Additional examples of high-resolution analysis include working with problems such as water and food security, dealing with infectious disease and heat, air quality long-term exposure assessments, and developing energy and other demand studies.

A data-driven decision narrative is often needed for cities to develop smart solutions as part of their operational efficiency, improved livability and short- and long-term resiliency outlooks. There is an increasing demand for high spatiotemporal resolution data over the urban regions for smart growth planning, emergency response, and management in response to the current changing climate (see Holden et al. (2011); Liu et al. (2020)). The rainfall and clouds over urban areas vary due to anthropogenic activities and changes in land use/land cover characteristics. In the study by Freitag et al. (2018), urban imprints were found in the precipitation and cloud processes in addition to changes caused to the upstream flow of water. Extreme rainfall over urban areas and particularly over urban-rural boundaries has shown increasing trends, and the signature can be found across the world (Freitag et al., 2018; Niyogi et al., 2017; Kishtawal et al., 2010). A resilient and sustainable response to the current and future climate scenarios relies on an accurate understanding of how climatic characteristics are modified by different sub-sections of a city. Accordingly, researchers have generated surface flux data at the sub-city scale resolutions (primarily for surface temperature and air quality) using different downscaling approaches (e.g., Agathangelidis and Cartalis (2019); Hofierka et al. (2020)).

### Urban precipitation downscaling

A downscaled urban precipitation product at high spatiotemporal scales is necessary to capture the different active processes. To circumvent the coarse-scale issue for impact and assessment studies, downscaling approaches have been employed (Abdollahipour et al., 2022). Downscaling operator improves the resolution of the coarse grid and sampling frequency datasets to higher resolution outputs. The operators used for such a transformation range from computationally expensive dynamical downscaling models (e.g., Leung and Qian (2005)) to the simpler two-dimensional linear interpolation (Shepard, 1968). Several statistical techniques have been applied in the literature related to cubic interpolation, kriging methods, random forests, support vector machines, artificial neural networks, and deep learning-based approaches (Sun & Tang, 2020; Sekulić et al., 2021; Sha et al., 2020; Sachindra et al., 2018; Wang et al., 2021). In the past, several studies have attempted urban precipitation downscaling. Sørup et al. (2016) downscale the regional climate model outputs using a statistical technique to a 2 km spatial resolution. Their goal is aimed towards urban hydrology and they use a dense network of station dataset over a limited region for the same. Ward et al. (2018) use a high-temporal resolution precipitation dataset to show that the difference in temporal resolution leads to improved modeled energy fluxes. Licznar et al. (2011) develop temporally high resolution data, Berne et al. (2004) show that high temporal resolution is required for high spatial resolutions for the urban hydrology applications. A study (Lu & Qin, 2014) downscaled the future climate projections on the stations over Singapore urban area by disaggregating and downscaling. Similar attempts have been made over Stockholm (Olsson et al., 2012) and Auckland (Akhter et al., 2019).

### Deep learning for urban precipitation downscaling

With the surge in recent deep learning/ machine learning interest, there is growing evidence that deep learning-based techniques can enhance the traditional statistical methods used to increase spatial resolution or downscaling of climate products (Tyagi et al. 2022). A number of studies, following the work by Dong et al. (2015), showed that a simple, lightweight image-to-image deep convolutional neural networks (SRCNN) can substantially outperform a widely used technique for spatial downscaling using two-dimensional cubic interpolation. Of interest also is that the simple deep learning-based solution is comparable to the sparse coding technique. Of particular interest to this study, is the issue of developing high resolution climate information over cities. Table 1 lists example of studies that have employed CNN-based algorithms (i.e., random forest, remote sensing indices, deep learning, support vector machine; artificial neural networks) to generate higher spatial resolution data over urban regions. Various studies (Xu et al., 2020; Choe & Yom, 2020; Xu et al., 2021; Hutengs & Vohland, 2016; Weng & Fu, 2014; Li et al., 2019; Yu & Liu, 2021; Liu et al., 2018; Sha et al., 2020) have explored downscaling variables important for urban areas. Examples include downscaling land surface temperature, air temperature, and air pollutants such as fine particulate matter (PM2.5), and nitrogen dioxide (NO2). The past studies (Table 1) have focused on machine learning methods such as random forests, kriging, support vector machines, and artificial neural networks. However, the application of deep learning is a recent approach.

**Table 1:** Comparison of machine learning / deep learning studies focused on urban downscaling

A	B	C	D	E	Scale-up	Study
**LST**	Guangzhou	RF			90 m to 10 m	Gu et al. (2015)
**LST**	Seoul	DL			1000 m to 30 m	Xu et al. (2020)
**Tb**	Guangzhou	RF			30 m to 10 m	Choe and Yom (2020)
**LST**	Zhangye	RS			270 m to 90 m	Xu et al. (2021)
**LST**	Jordan river valley	RF			1000m to 250 m	Pan et al. (2018)
**LST**	Los Angeles	SVM			5km to 1km	Hutengs and Vohland (2016)
**LST**	Beijing	SVM,RF,ANN			990m to 90m	Weng and Fu (2014)
**NO2**	Los Angeles	DL			0.125$^{\circ }$ to 5km	Li et al. (2019)
**PM2.5**	USA	RF+KR			0.1$^{\circ }$ to 0.01$^{\circ }$	Yu and Liu (2021)
**Tmax/Tmin**	USA	RF			0.25$^{\circ }$ to 4 km	Liu et al. (2018)
**T2m**	Tokyo	DL	$\checkmark$		10m to 5m	Sha et al. (2020)
**Pr**	Austin, Texas, USA	DL	$\checkmark$	$\checkmark$	10 km to 300 m	SRCNN (This study)

*A* variable (*LST* land surface temperature, *Tb* brightness temperature, *PM2.5* particulate matter smaller than 2.5 micron; *Pr* precipitation, *NO2* nitrogen, *Tmax/Tmin* maximum/minimum 2-m temperature, *T2m* two meter air temperature), *B* city/location (*GZ* Guangzhou, *ZH* Zhangye, *SL* Seoul, *JRV* Jordan river valley, *LA* Los Angeles, *BJ* Beijing), *C* (*RF* random forests, *RS* remote sensing indices, *DL* deep learning, *SVM* support vector machine, *ANN* artificial neural networks, *KR* kriging), *D* convolutional neural networks, *E* iterative downscaling

While most of these studies attempt to improve the spatial resolutions of the urban datasets, they do not employ CNN for high-resolution (<500 m) urban precipitation downscaling, which has shown superior performance on image-based tasks. Moreover, past studies have primarily focused on temperature and air pollution-related variables. High-resolution precipitation maps over urban regions, which are for non-continuous periods and with dynamic spatial heterogeneity have not been assessed by any study. Moreover, such high resolution precipitation data are important for are important for urban hydrology applications. Also, most studies, as shown in Table 1, have attempted at the downscaling factors of up to 10x, except for the work done by Xu et al. (2020), who attempt to downscale up to 30x of the low-resolution inputs.

### Study contributions

We focus on the problem of precipitation downscaling over urban areas using the deep learning/SRCNN approach using Austin, Texas, USA, as the urban domain. High-resolution sub-500 m datasets of precipitation required by the City of Austin are not yet available by any available product. While applications of CNN-based methods have resulted in satisfactory outcomes, the networks of these algorithms are rather complex. Super-Resolution Convolutional Neural Network (SRCNN) (Dong et al., 2015) is a simple, lightweight network structure with a high restoration quality. Given the potential of the SRCNN method, we postulate that high-quality rainfall data with fine spatial resolution could be generated using iterative SRCNN over the urban region with higher accuracy and speed than standard CNN methods. We test this approach because downscaling rainfall data over the urban areas is particularly challenging as the rainfall characteristics are modified by the city’s microclimate (Freitag et al., 2018). The heterogeneous environment of urban regions with varying physical and thermodynamic properties and anthropogenic activities alters the surface flux and impacts the atmospheric boundary layer, which ultimately translates into a shift in rainfall regime over the urban landscape (Onishi et al., 2019). We postulate that urban precipitation can be downscaled to very-high resolution over Austin, Texas by ingesting data from the Japan Aerospace Exploration Agency (JAXA) satellite product as a precursor to the deep learning model. Our method is expected to create an ability for generating fast, high spatiotemporal meteorological datasets with super-resolution that have significant implications for the current and future development plans of urban climate services and smart cities.

In this study, we employ a deep CNN-based model - SRCNN to iteratively downscale the precipitation data over Austin, Texas from JAXA global product available at 0.1 $^{\circ }$ hourly spatiotemporal resolutions from 2000 to 2020. We take the reference of a general purpose statistical downscaling operator such as cubic or linear interpolation, which can theoretically increase the resolution to generate finer-scale datasets. The task performed by the traditional statistical operators is to rearrange the low-resolution information into a dense matrix. We attempt a similar transformation and develop the iterative SRCNN to perform 2x downscaling at any scale, i.e., from 10km to 5km, from 5km to 2.5 km and so on.

Thus, our contributions include the development of a general-purpose algorithm based on a single image super-resolution in computer vision by using sub-images to perform iterative downscaling for high-resolution urban-scale datasets. Our approach can be used to iteratively generate downscaled products, theoretically up to any spatial resolution for which images are available. As stated, we focus on achieving spatial resolutions of $\sim$ 300 meters. This spatial resolution can be used for targeted applications to communities planning climate resiliency and adaptation strategies for urban neighborhoods. Generating $\sim$ 300-meter precipitation maps from measurements is challenging as it would require the deployment of measurement sensors in large quantities. The initial setup of such sensors and their regular maintenance also would be quite expensive and even more challenging for maintaining and operating. The iterative method provides a workaround for traditional techniques by generating high-resolution urban precipitation datasets. We target a scaling factor of around 30x from input low-resolution satellite product at 0.1 degrees or 10km to a target high-resolution output of $\sim$ 300 meters.

## Data and methodology

**Fig. 2:**
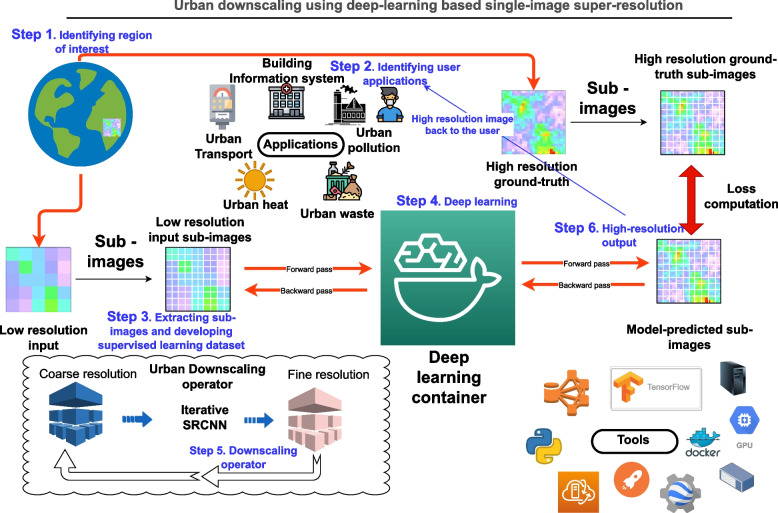
Schematic showing the process used to downscale the rainfall dataset over Austin, Texas, USA using Iterative SRCNN in this study. Step 1 to Step 6 show the process used to develop high-resolution output

This section discusses the data and methodology used in this study. The schematic of the data and methodology, in addition to the applications of high-resolution urban climate datasets is shown in Fig. 2.

### Dataset

The Japan Aerospace Exploration Agency (JAXA) Global Rainfall Watch (GsMAP), which is a part of the Global Precipitation Measurement (GPM) is used in this study. The state-of-the-art Ku/Ka Doppler dual-frequency precipitation radar (DPR) and microwave imager are aboard GPM’s primary satellite. Because of the improvements made by its load over TRMM in identifying tropical precipitation (0-1 mm per day) (Draper et al., 2015), GPM provides high quality global satellite precipitation measurements. The setup also includes dual-polarized doppler radar based output which can give very high-resolution precipitation data. GPM consists of two algorithms for satellite-based precipitation. One of them is the IMERG (Hou et al., 2014) from NASA, and the other is GsMAP (Kubota et al., 2007) from JAXA. JAXA GsMAP has various products in its catalog: near real-time, moving vector with Kalman filter, and the gauge-calibrated standard product.

In this study, we use the JAXA GsMap gauge-calibrated precipitation product. The dataset provides global coverage and is available from 2000 to the present at a spatial resolution of 0.1$^{\circ }$ and a temporal resolution of one hour. A subdomain of JAXA data encompassing Austin, Texas and covering 29 - 32N, 96 - 99W was selected. Although a large-scale dataset over the Earth needs to consider sphericity, our dataset is over a small region relative to the global dataset from which it is acquired. Because of the smaller domain size, it is considered a two-dimensional image. The algorithms applicable to two-dimensional images in computer vision are therefore considered suitable for this data. The data is first split into training and testing data, with the training data corresponding to 2001 to 2009 and test data as 2010 to 2018. The domain over Austin is selected as a 3 $^{\circ }$ x 3$^{\circ }$ box, and the spatial resolution of JAXA GsMAP data is 0.1 $^{\circ }$ x 0.1 $^{\circ }$. Thus, the original data is a matrix of size 30 x 30.

### Methodology

The training data is normalized using min-max scaling and then transformed to sub-images of size 20 $\times$ 20. The test dataset is scaled using the normalization weights from the training data. The sub-images are then fed into the SRCNN algorithm. The deep convolutional neural network employs the following equations:

1 $$\begin{aligned} F1(Y) = max(0, W1 * Y + B1) \end{aligned}$$

2 $$\begin{aligned} F2(Y) = max(0, W2 * F1(Y) + B2) \end{aligned}$$

3 $$\begin{aligned} F3(Y) = max(0, W3 * F2(Y) + B3) \end{aligned}$$

Here, Y is the input coarse-resolution dataset and F3(Y) is the output high-resolution output. W1, W2, W3 and B1, B2, B3 correspond to the weights and biases learned during training. The non-linear function used is a rectified linear unit which is max(0,x). The architectural details of the model can be seen in detail from Dong et al. (2015). First, the 20 $\times$ 20 sub-images are convoluted by 64 filters, each with a size 9 $\times$ 9, and then the rectified linear unit (RELU) activation function is operated upon the convolutions. This operation involves the convolution and non-linear activation functions described by Eq. 1. The output of Eq. 1 again goes through the transformation involving convolution and activation; however, 32 filters of size 1 $\times$ 1 complete the operations of Eq. 2. The output of Eq. 2 is then operated by one 5 $\times$ 5 filter to perform a linear combination operation which is the output of Eq. 3. We use the padding option ‘same’ so that the operations are padded and the input and output size remains identical. The applied optimizer is adaptive moment estimation (ADAM) with a learning rate of 0.001. The model is trained from 2001 to 2008, with the year 2009 corresponding to the validation period during training. The best model is saved every 100 iterations if the validation loss falls below the previous best model. Once the training is completed, the test data is first normalized and broken down into sub-images to be fed as input to the trained model. The test predictions are then reconstructed from the sub-images and inverse normalized to compare with the ground truth in the test dataset. The model is a general purpose operator that is capable of performing twofold super-resolution from any low-resolution information to a higher resolution matrix.

In a nutshell, the output from SRCNN at 10 km spatial resolution is used as an input to the trained model to generate an output at 5 km in an iterative manner. Iterative training is possible as we train the model using sub-images which is the core of our algorithm. Hence the model is agnostic to the size of the input dataset, and any large image can be broken into sub-images of size 20 x 20. These sub-images are reconstructed back after the model predictions. This property of our deep learning-based solution makes the model comparable to a standard interpolation technique such as cubic interpolation. Thus, it is subsequently used in an iterative framework to generate the urban scale ($\sim$ 300 m) product from 10 km JAXA GsMAP satellite precipitation product. The complete training with early stopping took around one week on an NVIDIA Tesla P100 GPU. The super-resolution downscaled datasets are generated in an hour from the trained model for ten years of testing data.

### Need for iterative SRCNN

Iterative SRCNN is used in this urban downscaling, and is the backbone of this study. In general, the SRCNN is a general-purpose operator which can perform a 2x downscaling from the 10 km resolution GsMAP precipitation data using single-image super-resolution. The idea is similar to the general-purpose operators such as bilinear/cubic interpolation which can perform interpolation up or down the scale. However, we choose to perform iterative downscaling transcending one step at a time as in spatial scales as it preserves the information squeezing rather than taking the big leap forward.

### Limitations of iterative SRCNN

There are different statistical approaches are available for such a downscaling framework. Each have their strengths and limitations (Kotamarthi et al., 2021). The utility of SRCNN as a data resolution operator is well established in the vision community (Yamanaka et al., 2017). In general, SRCNN is a good first choice for this DownScaleWorkbench, because of its robustness, wide usage, and computational efficiency. Nonetheless, as with any technique, there are important limitations that are highlighted here. The first shortcoming is the emergence of horizontal and vertical lines, which occur as artifacts when downscaling is performed beyond a certain spatial resolution. At what resolution this effect would become visible can only be determined by experimentation (Song et al., 2019). Second, the method only considers spatial information, whereas localized heavy extremes have temporal disaggregation (Scher & Peßenteiner, 2021), which is not considered in this study. In terms of the general limitations of this study, which is often the case with many downscaling exercises, is that lack of reference high-resolution dataset to validate the generated values. In an experimental setting, this can be conducted by setting up rain-gauges over a limited area to generate a very high-resolution gridded product to validate the output of Iterative SRCNN. Moreover, hyper-local convective rain events are averaged over the whole grid in the input data, and this method may not be able to identify and localize such events. Therefore, such a method likely works best for larger scale frontal rain events to improve the spatial information at a local scale.

## Development of DownScaleBench

**Fig. 3:**
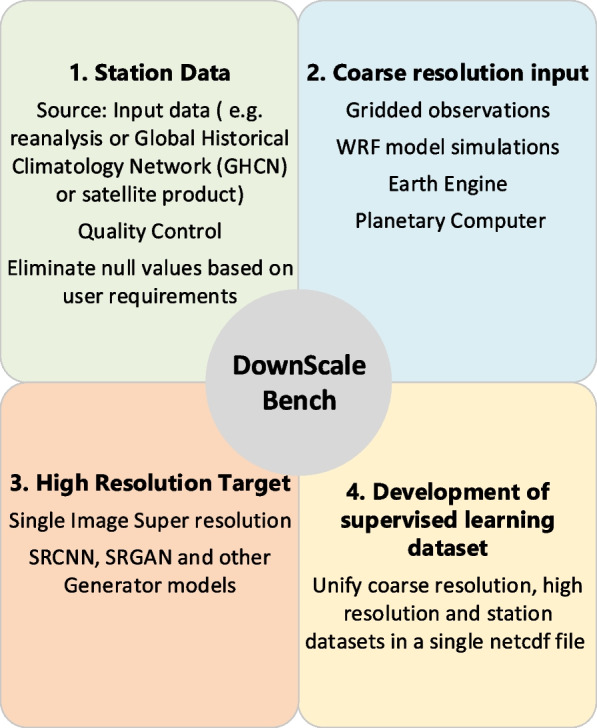
Components of the DownScaleBench framework for generating supervised learning datasets for urban downscaling. An integral part of DownScaleBench is the incorporation of station information in the training process

A predetermined sequence of operations is required to produce the DownScaleBench. This workflow is also shown in Fig. 3. Initially, we define the area of interest and then obtain data from a climate dataset such as the Global Historical Climatology Network (GHCN). From the GHCN dataset, the ground station data available within a 3 deg x 3 deg (  300 km x 300 km) box that surrounds the area of interest is extracted. This choice of the 300 km grid box is based on consideration of data availability, data quantity, and the computational aspects such as data storage needs. This domain size is also considered based on the typical size of cities globally and the rainfall feedback that is expected over a region that is typically twice the size of the city (Liu & Niyogi, 2019; Niyogi et al., 2011).

Second step of the DownScaleBench is to undertake quality control on the station data by removing stations with excessive null values or with large missing data. This criterion can be user-specified and in our study, we eliminated stations which had data missing for multiple years. We identify the stations with the lowest null values and the longest period of records. These stations are used for further analyses.

Third, we have the option and the ability of extracting the gridded products currently available corresponding to the domain and variable of interest using Google Earth Engine.

Finally, we can extract the ground station data during the period where both the gridded products and station data are available in the database. The data from the ground stations and the gridded product are brought together within the DownScaleBench.

Thus, DownScaleBench addresses the issue of standardizing the validation and access of the high-resolution climate data over and around the city from ground stations as well as gridded products and provide an interface for ingesting these data for the training of the ML algorithms. The Jupyter notebook to run DownScaleBench is provided with the GitHub repository (https://github.com/texuslabut/urban_precipitation_downscaling) for this study.

## Results and discussion

The test predictions generated for 2010 to 2019 are compared with the ground truth data for the same period. The ground truth data considered is the same as the original JAXA GsMAP gridded dataset over Austin at 10 km spatial resolution. Any hourly data that report ‘no rainfall’ in the input is masked from the metrics used to compare the cubic interpolation baseline and SRCNN-based deep learning model. Two metrics, viz, Peak Signal to Noise Ratio (PSNR) and Mutual Information, are used to compare the test predictions relative to the ground truth (the JAXA GsMAP satellite product). PSNR quantifies the ratio of a highest potential signal strength to its confounding noise power. It is measured by the decibel scale. PSNR is a typical metric for gauging the quality of an image. Compression introduces pixel-level errors into the data and PSNR is considered an approximation of human perception of reconstruction quality when comparing compression codecs (Li & Cai, 2007). As a result, PSNR is a useful metric to compare pixel or grid-level errors in two images or matrices. Mutual information is the other metric used. It is an advanced nonlinear index measuring the similarity of distributions (Speed, 2011). The PSNR increases from 146.96 in the baseline to 149.46 for SRCNN compared with the original 10 km dataset. To understand whether the improvement is notable (significant) or not, we compare the results against the survey of different metric presented in Wang et al. (2019). Accordingly, this increase in the PSNR by 2.5 is a strong indicator of the enhancement achieved. The improvement is also noted in the mutual information results from 0.59 (in the baseline) to 0.62.

**Fig. 4:**
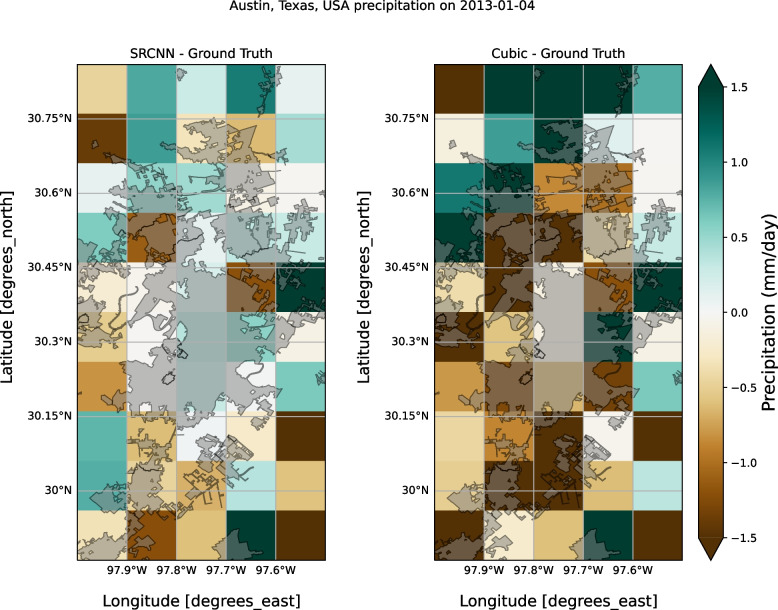
Difference in precipitation maps as cubic interpolation minus the JAXA GsMAP satellite rainfall, and SRCNN minus the JAXA GsMAP satellite rainfall

A spatial comparison of SRCNN prediction and the cubic interpolation baseline with the ground truth is shown in Fig. 4, for a heavy rainfall case that occurred 4 January 2013 daily precipitation. These matrices show improved pixel-level information in SRCNN. Although our algorithm shows pixel level improvements of up to 2 mm/day at the resolution of the input satellite data, the high-resolution precipitation product is a matrix of size 30x higher (finer) resolution relative to the input satellite data (10 km). Errors typically average out and reduce for larger grid sizes. That is the error or bias over the larger 10 km X 10 km grid would exponentially grow to a large bias in a concentrated gridded product of grid size 300 m X 300 m. Iterative forecasts are generated up to 300 m and beyond 5 km iterative forecasts. A more detailed spatial structure is noted in the downscaled urban rainfall as shown in Fig. 3. Verifying the higher resolution output is challenging, especially because of the lack of reference data at comparable resolution.

**Fig. 5:**
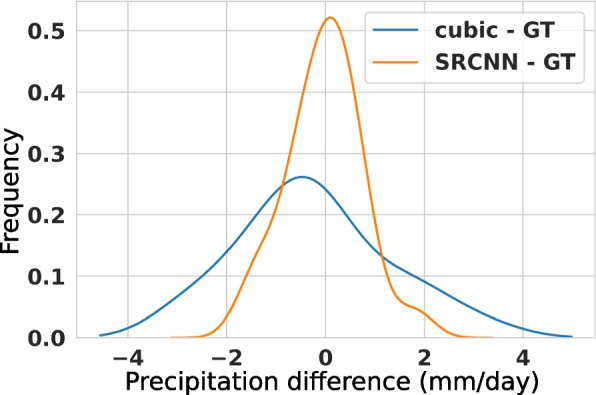
Distributions of cubic minus ground truth (GT) and SRCNN minus ground truth (GT) show under and overestimation of precipitation in cubic interpolation baseline

Figure 5 reviews the distribution of difference between JAXA 10km rainfall as the reference and the difference obtained by cubic and SRCNN downscaling. The plot shows an under and over-representation of the pixel-level rainfall that can be observed in the cubic interpolation relative to SRCNN. Cubic interpolation does not transform and only interpolates, while the SRCNN provides a finer spatial outcome.

**Fig. 6:**
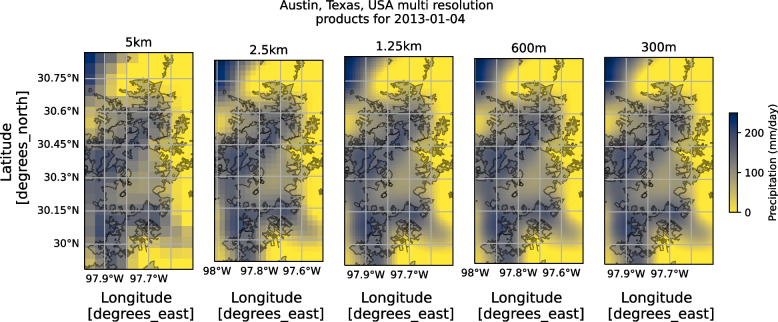
Multi-resolution maps of the gridded rainfall product over Austin using Iterated SRCNN

Figure 6 shows the resulting final products using iterative SRCNN for different spatial resolutions. These maps are restricted to a stable downscaled product at 300 m X 300 m gridded spatial resolution. The stability was based on the consideration of the point at which horizontal and vertical discontinuity lines appeared in the downscaled output from deep learning. These discontinuities are an artifact of SRCNN as discussed in previous studies (Song et al., 2019). In future studies, advanced deep learning-based super-resolution methodologies for downscaling can likely help eliminate these distortions and will be explored. By reviewing Fig. 6, it can be visually noted that the rainfall fields become more continuous and smoother as the resolution increases.

## Conclusions and future work

This study successfully employs a deep learning-based convolutional neural network, SRCNN, for urban downscaling. High-resolution maps of precipitation at 300 m were generated from the 10 km grid resolution satellite-based JAXA GsMAP product over Austin, Texas (29-32N, 96-99W). The deep learning-based model is trained for 2001 to 2009, while the test predictions are generated for 2010 to 2019. The quantitative metrics, as well as visual inspection, show substantial improvements in the pixel-level information from the iterative SRCNN downscaling relative to the baseline cubic interpolation. The highest resolution dataset at 300 meters is generated by using iterative prediction. Efforts to improve the dataset in the future include training the model using hyperparameter tuning as a follow-up study. Increasing the number of layers and using residual or generative networks will also be explored. A study underway for the soil moisture dataset with this method includes taking the coarse-resolution satellite data as an input and developing a high-resolution output with reduced latency. This study through the development of DownScaleBench provides an efficient framework with low computational cost and higher speed for generating high-resolution data over urban regions. The workflow outlined in DownScaleBench will also help standardize the comparison (verification) of high-resolution gridded urban datasets, which was challenging due to the absence of a baseline and an adopted framework to incorporate the ground station data.

Such data, it is anticipated, could be used by city decision-makers for sustainable developments. The downscaled climate products can be generated within minutes after training for one image. To the best of our knowledge, this is the first study to perform urban downscaling for precipitation datasets. The increasing trend of heavy rainfall and localized floods over urban areas make this high-resolution spatiotemporal dataset useful to identify potentially vulnerable areas and address the infrastructural requirements of those regions (Bixler et al., 2022). Advanced deep learning algorithms using attention and generative learning provide good solutions to improve these high-resolution products further (Singh et al., 2022).

## Data Availability

The datasets and materials used in the study are open source and in public domain.

## References

[CR1] Abdollahipour A, Ahmadi H, Aminnejad B (2022). A review of downscaling methods of satellite-based precipitation estimates. Earth Science Informatics.

[CR2] Agathangelidis I, Cartalis C (2019). Improving the disaggregation of modis land surface temperatures in an urban environment: a statistical downscaling approach using high-resolution emissivity. International Journal of Remote Sensing.

[CR3] Akhter MS, Shamseldin AY, Melville BW (2019). Comparison of dynamical and statistical rainfall downscaling of cmip5 ensembles at a small urban catchment scale. Stochastic Environmental Research and Risk Assessment.

[CR4] Aloysius, N. & Geetha, M. (2017). A review on deep convolutional neural networks. In *2017 International Conference on Communication and Signal Processing (ICCSP)* (pp. 0588–0592). IEEE.

[CR5] Anthopoulos L (2017). Smart utopia vs smart reality: Learning by experience from 10 smart city cases. Cities.

[CR6] Berne A, Delrieu G, Creutin J-D, Obled C (2004). Temporal and spatial resolution of rainfall measurements required for urban hydrology. Journal of Hydrology.

[CR7] Bixler, R. P., Coudert, M., Richter, S. M., Jones, J. M., Pulido, C. L., Akhavan, N., Bartos, M., Passalacqua, P., & Niyogi, D. (2022). Reflexive co-production for urban resilience: Guiding framework and experiences from austin, texas. *Frontiers in Sustainable Cities*, *4*, 178.

[CR8] Choe Y-J, Yom J-H (2020). Improving accuracy of land surface temperature prediction model based on deep-learning. Spatial Information Research.

[CR9] Dong C, Loy CC, He K, Tang X (2015). Image super-resolution using deep convolutional networks. IEEE Transactions on Pattern Analysis and Machine Intelligence.

[CR10] Draper DW, Newell DA, Wentz FJ, Krimchansky S, Skofronick-Jackson GM (2015). The global precipitation measurement (gpm) microwave imager (gmi): Instrument overview and early on-orbit performance. IEEE Journal of Selected Topics in Applied Earth Observations and Remote Sensing.

[CR11] Freitag B, Nair U, Niyogi D (2018). Urban modification of convection and rainfall in complex terrain. Geophysical Research Letters.

[CR12] Ghosh, S. (2010). Svm-pgsl coupled approach for statistical downscaling to predict rainfall from gcm output. *Journal of Geophysical Research: Atmospheres*, *115*(D22). https://agupubs.onlinelibrary.wiley.com/toc/21562202d/2010/115/D22.

[CR13] González JE, Ramamurthy P, Bornstein RD, Chen F, Bou-Zeid ER, Ghandehari M, Luvall J, Mitra C, Niyogi D (2021). Urban climate and resiliency: A synthesis report of state of the art and future research directions. Urban Climate.

[CR14] Gu, J., Wang, Z., Kuen, J., Ma, L., Shahroudy, A., Shuai, B., Liu, T., Wang, X., & Wang, G. (2015). Recent advances in convolutional neural networks. eprint. arXiv preprint arXiv:1512.07108.

[CR15] Hersbach H, Bell B, Berrisford P, Hirahara S, Horányi A, Muñoz-Sabater J, Nicolas J, Peubey C, Radu R, Schepers D (2020). The era5 global reanalysis. Quarterly Journal of the Royal Meteorological Society.

[CR16] Hofierka J, Gallay M, Onačillová K, Hofierka J (2020). Physically-based land surface temperature modeling in urban areas using a 3-d city model and multispectral satellite data. Urban Climate.

[CR17] Holden ZA, Abatzoglou JT, Luce CH, Baggett LS (2011). Empirical downscaling of daily minimum air temperature at very fine resolutions in complex terrain. Agricultural and Forest Meteorology.

[CR18] Hou AY, Kakar RK, Neeck S, Azarbarzin AA, Kummerow CD, Kojima M, Oki R, Nakamura K, Iguchi T (2014). The global precipitation measurement mission. Bulletin of the American Meteorological Society.

[CR19] Hu H, Hu Z, Zhong K, Xu J, Zhang F, Zhao Y, Wu P (2019). Satellite-based high-resolution mapping of ground-level pm2. 5 concentrations over east china using a spatiotemporal regression kriging model. Science of The Total Environment.

[CR20] Hutengs C, Vohland M (2016). Downscaling land surface temperatures at regional scales with random forest regression. Remote Sensing of Environment.

[CR21] Kishtawal CM, Niyogi D, Tewari M, Pielke RA, Shepherd JM (2010). Urbanization signature in the observed heavy rainfall climatology over india. International journal of climatology.

[CR22] Kotamarthi, R., Hayhoe, K., Wuebbles, D., Mearns, L. O., Jacobs, J., & Jurado, J. (2021). *Downscaling techniques for high-resolution climate projections: From global change to local impacts*. Cambridge University Press.

[CR23] Kubota T, Shige S, Hashizume H, Aonashi K, Takahashi N, Seto S, Hirose M, Takayabu YN, Ushio T, Nakagawa K (2007). Global precipitation map using satellite-borne microwave radiometers by the gsmap project: Production and validation. IEEE Transactions on Geoscience and Remote Sensing.

[CR24] Leung, L.-Y. R. & Qian, Y. (2005). Downscaling extended weather forecasts for hydrologic prediction. *Bulletin of the American Meteorological Society*, *86*(PNWD-SA-6940).

[CR25] Li W, Ni L, Li Z-L, Duan S-B, Wu H (2019). Evaluation of machine learning algorithms in spatial downscaling of modis land surface temperature. IEEE Journal of Selected Topics in Applied Earth Observations and Remote Sensing.

[CR26] Li, X. & Cai, J. (2007). Robust transmission of jpeg2000 encoded images over packet loss channels. In *2007 IEEE International Conference on Multimedia and Expo* (pp. 947–950). IEEE.

[CR27] Licznar P, Łomotowski J, Rupp DE (2011). Random cascade driven rainfall disaggregation for urban hydrology: An evaluation of six models and a new generator. Atmospheric Research.

[CR28] Liu J, Niyogi D (2019). Meta-analysis of urbanization impact on rainfall modification. Scientific Reports.

[CR29] Liu X, Huang Y, Xu X, Li X, Li X, Ciais P, Lin P, Gong K, Ziegler AD, Chen A (2020). High-spatiotemporal-resolution mapping of global urban change from 1985 to 2015. Nature Sustainability.

[CR30] Liu Y, Cao G, Zhao N, Mulligan K, Ye X (2018). Improve ground-level pm2. 5 concentration mapping using a random forests-based geostatistical approach. Environmental Pollution.

[CR31] Lu Y, Qin X (2014). Multisite rainfall downscaling and disaggregation in a tropical urban area. Journal of Hydrology.

[CR32] Meehl GA, Boer GJ, Covey C, Latif M, Stouffer RJ (2000). The coupled model intercomparison project (cmip). Bulletin of the American Meteorological Society.

[CR33] Navon, I. M. (2009). Data assimilation for numerical weather prediction: a review. *Oceanic and Hydrologic Applications: Data assimilation for Atmospheric*, 21–65.

[CR34] Niyogi D, Lei M, Kishtawal C, Schmid P, Shepherd M (2017). Urbanization impacts on the summer heavy rainfall climatology over the eastern united states. Earth Interactions.

[CR35] Niyogi D, Pyle P, Lei M, Arya SP, Kishtawal CM, Shepherd M, Chen F, Wolfe B (2011). Urban modification of thunderstorms: An observational storm climatology and model case study for the indianapolis urban region. Journal of Applied Meteorology and Climatology.

[CR36] Njoku EG, Wilson WJ, Yueh SH, Dinardo SJ, Li FK, Jackson TJ, Lakshmi V, Bolten J (2002). Observations of soil moisture using a passive and active low-frequency microwave airborne sensor during sgp99. IEEE Transactions on Geoscience and Remote Sensing.

[CR37] Olsson J, Willén U, Kawamura A (2012). Downscaling extreme short-term regional climate model precipitation for urban hydrological applications. Hydrology Research.

[CR38] Onishi, R., Sugiyama, D., & Matsuda, K. (2019). Super-resolution simulation for real-time prediction of urban micrometeorology. *SOLA, 15*, 178-182. Meteorological Society of Japan.

[CR39] Pan X, Zhu X, Yang Y, Cao C, Zhang X, Shan L (2018). Applicability of downscaling land surface temperature by using normalized difference sand index. Scientific Reports.

[CR40] Sachindra D, Ahmed K, Rashid MM, Shahid S, Perera B (2018). Statistical downscaling of precipitation using machine learning techniques. Atmospheric Research.

[CR41] Scher S, Peßenteiner S (2021). Temporal disaggregation of spatial rainfall fields with generative adversarial networks. Hydrology and Earth System Sciences.

[CR42] Schumacher RS, Rasmussen KL (2020). The formation, character and changing nature of mesoscale convective systems. Nature Reviews Earth & Environment.

[CR43] Sekulić A, Kilibarda M, Protić D, Bajat B (2021). A high-resolution daily gridded meteorological dataset for serbia made by random forest spatial interpolation. Scientific Data.

[CR44] Sha Y, Gagne DJ, West G, Stull R (2020). Deep-learning-based gridded downscaling of surface meteorological variables in complex terrain. part i: Daily maximum and minimum 2-m temperature. Journal of Applied Meteorology and Climatology.

[CR45] Shepard, D. (1968). A two-dimensional interpolation function for irregularly-spaced data. In *Proceedings of the 1968 23rd ACM national conference* (pp. 517–524).

[CR46] Singh, M., Acharya, N., Rao, S. A., Kumar, B., Yang, Z.-L., Niyogi, D., et al. (2022). Short-range forecasts of global precipitation using deep learning-augmented numerical weather prediction. *arXiv e-prints*, pages arXiv–2206.

[CR47] Smid M, Costa AC (2018). Climate projections and downscaling techniques: a discussion for impact studies in urban systems. International Journal of Urban Sciences.

[CR48] Song, R. et al. (2019). Improved super-resolution convolution neural network for large images. arXiv preprint arXiv:1907.12928.

[CR49] Sørup HJD, Christensen OB, Arnbjerg-Nielsen K, Mikkelsen PS (2016). Downscaling future precipitation extremes to urban hydrology scales using a spatio-temporal neyman-scott weather generator. Hydrology and Earth System Sciences.

[CR50] Speed T (2011). A correlation for the 21st century. Science.

[CR51] Sun, A. Y. & Tang, G. (2020). Downscaling satellite and reanalysis precipitation products using attention-based deep convolutional neural nets. *Frontiers in Water*, 56.

[CR52] Tewari K, Tewari M, Niyogi D (2023). Need for considering urban climate change factors on stroke, neurodegenerative diseases, and mood disorders studies. Computational Urban Science.

[CR53] Wang F, Tian D, Lowe L, Kalin L, Lehrter J (2021). Deep learning for daily precipitation and temperature downscaling. Water Resources Research.

[CR54] Wang, Z., Chen, J., & Hoi, S. C. (2019). Deep learning for image super-resolution: A survey. arXiv preprint arXiv:1902.06068. 10.1109/TPAMI.2020.2982166 32217470

[CR55] Ward H, Tan Y, Gabey A, Kotthaus S, Grimmond CB (2018). Impact of temporal resolution of precipitation forcing data on modelled urban-atmosphere exchanges and surface conditions. International Journal of Climatology.

[CR56] Weng Q, Fu P (2014). Modeling diurnal land temperature cycles over los angeles using downscaled goes imagery. ISPRS Journal of Photogrammetry and Remote Sensing.

[CR57] Xu J, Zhang F, Jiang H, Hu H, Zhong K, Jing W, Yang J, Jia B (2020). Downscaling aster land surface temperature over urban areas with machine learning-based area-to-point regression kriging. Remote Sensing.

[CR58] Xu J, Zhang F, Ruan H, Hu H, Liu Y, Zhong K, Jing W, Yang J, Liu X (2021). Hybrid modelling of random forests and kriging with sentinel-2a multispectral imagery to determine urban brightness temperatures with high resolution. International Journal of Remote Sensing.

[CR59] Yamanaka, J., Kuwashima, S., & Kurita, T. (2017). Fast and accurate image super resolution by deep cnn with skip connection and network in network. In *Neural Information Processing: 24th International Conference, ICONIP 2017, Guangzhou, China, November 14-18, 2017, Proceedings, Part II 24* (pp. 217–225). Springer.

[CR60] Yang L, Smith J, Baeck ML, Smith B, Tian F, Niyogi D (2016). Structure and evolution of flash flood producing storms in a small urban watershed. Journal of Geophysical Research: Atmospheres.

[CR61] Yu M, Liu Q (2021). Deep learning-based downscaling of tropospheric nitrogen dioxide using ground-level and satellite observations. Science of The Total Environment.

